# Control over Conflict during Movement Preparation: Role of Posterior Parietal Cortex

**DOI:** 10.1016/j.neuron.2008.02.009

**Published:** 2008-04-10

**Authors:** Elizabeth J. Coulthard, Parashkev Nachev, Masud Husain

**Affiliations:** 1 Institute of Cognitive Neuroscience and Institute of Neurology, University College London, London WC1N 3AR, UK; 2 Imperial College London, Charing Cross Campus, The Reynolds Building, St. Dunstans's Road, Hammersmith, London W6 8RP, UK

**Keywords:** SYSNEURO

## Abstract

Flexible behavior in humans often requires that rapid choices be made between conflicting action plans. Although much attention has focused on prefrontal regions, little is understood about the contribution of parietal cortex under situations of response conflict. Here we show that right parietal damage associated with spatial neglect leads to paradoxical facilitation (speeding) of rightward movements in the presence of conflicting leftward response plans. These findings indicate a critical role for parietal regions in action planning when there is response competition. In contrast, patients with prefrontal damage have an augmented cost of conflict for both leftward and rightward movements. The results suggest involvement of two independent systems in situations of response conflict, with right parietal cortex being a crucial site for automatic activation of competing motor plans and prefrontal regions acting independently to inhibit action plans irrelevant to current task goals.

## Introduction

Engaging in successful behavior requires animals to select appropriate actions in highly variable situations. If the response is invariantly defined by the stimulus or environmental context, there is no difficulty in selection. Frequently, however, there is more than one possible action choice. Under these circumstances, there is potential conflict between response plans and it is necessary for brain mechanisms to select the best response to achieve the animal's goal.

Although most studies have focused on the role of prefrontal regions (Botvinick et al., 2004; Egner and Hirsch, 2005; Garavan et al., 2003; Gehring and Fencsik, 2001; Nachev et al., 2005; Ridderinkhof et al., 2004; Rushworth et al., 2004; Ullsperger and von Cramon, 2001; van Veen et al., 2001), it is clear that conflicting potential responses evoked by the stimulus environment are also associated with parietal activity (Bunge et al., 2002; Liston et al., 2006; Stoet and Snyder, 2007). However, the role of posterior parietal cortex (PPC) in situations of conflict has not been extensively studied. Indeed, because previous studies have examined only activity in intact PPC, and not what occurs following lesions to this region, it remains to be established if the PPC is necessary for behavior under these circumstances.

We hypothesized that the PPC plays an important role in the selection of action under situations of response conflict, when stimulus-evoked responses activate conflicting action plans. In humans, damage to the PPC, most prominently in the right hemisphere, often leads to the syndrome of unilateral neglect, in which patients tend to be unaware of objects to their left (Bartolomeo et al., 2007; Doricchi and Tomaiuolo, 2003; Hillis et al., 2005; Humphreys and Riddoch, 2001; Mort et al., 2003; Robertson, 2003). In addition to perceptual and attentional factors that contribute to neglect of leftward items (Driver and Mattingley, 1998; Duncan et al., 1997; Husain and Rorden, 2003; Mesulam, 1999), some investigators have also reported directional motor deficits resulting in delayed reaching to contralesional objects—directional hypokinesia (DH)—in patients with neglect following either parietal or frontal lesions (Behrmann and Meegan, 1998; Coulthard et al., 2006; Ladavas et al., 1993; Mattingley et al., 1998; Sapir et al., 2007). However, the role of the PPC in motor control has been highly contentious and no clear consensus has emerged from studies in either humans or monkeys. Thus, while some authors have presented data in support of a key role in programming spatially directed action (Battaglia-Mayer et al., 2003; Gail and Andersen, 2006; Milner and Goodale, 1995; Rushworth et al., 2003; Snyder et al., 1997; Wascher et al., 1999), others have argued that these findings may be explained by the visual or attentional functions of the PPC (Colby and Goldberg, 1999; Goldberg et al., 2006; Gottlieb, 2007; Wardak et al., 2004).

**Figure 1:**
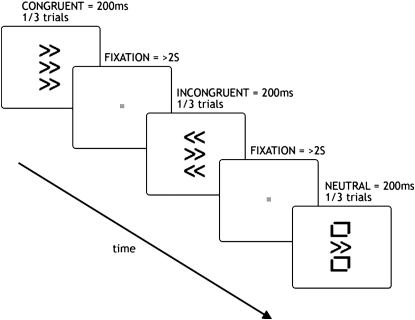
Directional Eriksen Flanker Task Using Vertical Arrays Subjects made a speeded response left or right using a small joystick placed centrally. Central arrows were flanked by arrows in the same (congruent) or opposite (incongruent) direction or by squares (neutral condition). The order of stimuli was pseudorandomized with the constraint that the same number of each condition occurred in each block. After each response, there was a delay of 2 s before the next stimulus was presented, ensuring that the interstimulus interval was at least 2 s.

To investigate our hypothesis that one important role of the PPC might be selection of action when stimulus-evoked responses activate conflicting action plans, we sought to examine whether response conflict might influence directional movement in PPC patients with neglect. Many studies of conflict in healthy individuals use variations of the Eriksen flanker task (Botvinick et al., 1999; Bunge et al., 2002; Eriksen and Eriksen, 1974; Scerif et al., 2006; Ullsperger and von Cramon, 2004, 2006; Ullsperger et al., 2002), in which responses to a central cue, e.g., an arrow instructing one movement, are delayed if it is flanked by incongruent stimuli, e.g., arrows in the opposite direction (Figure 1). This increase in reaction time (RT) is considered to index interference from competing neural responses evoked by cue and flankers in sensorimotor representations, where sensory cues (in this case, arrows) map to motor responses (movement direction) (Eriksen, 1995). Most discussions of this phenomenon consider the RT “cost” as a feature that should optimally be suppressed if subjects are to make rapid responses. In predictable circumstances, simple “rules” might be applied at early stages of processing to eliminate the effect of competing responses from the central cue and peripheral, irrelevant flankers in the Eriksen task. However, although the cost evoked by flankers is modifiable (Mayr et al., 2003), it is never to our knowledge completely eliminated, suggesting that competition is a robust process or is even perhaps hardwired to occur within our nervous systems.

Movement delay, therefore, is the result of competition between alternative responses. But rather than considering this simply as an inevitable cost, the delay evoked by conflict might actually also be functionally important, allowing selection between competing action choices before the response is made. For an animal, it might be worth paying the penalty of a small increase in RT (evoked by such conflict) to ensure that the most appropriate response is made. Even if some potential action choices are often irrelevant, there may be occasions when they represent the best response, particularly in natural, unpredictable environments. For example, a sudden change in the luminance of the visual scene may require very different responses depending upon the cause: if it is simply due to shadows cast by clouds, we may be able to ignore this and continue with the task at hand, but if it is due to falling masonry or bricks, we need to take aversive action rapidly. Here two action plans are potentially in conflict and the brain has to make a decision, based on prior probabilities and accumulating evidence, on which to select. Thus, although the competition evoked by flanker stimuli in the Eriksen paradigm is always irrelevant, this would not invariably be the case for stimuli in the real world.

According to this view, therefore, both relevant and irrelevant competing stimulus-response association signals propagate in the brain, mutually inhibiting each other and leading to an RT delay. Indeed, many current decision-making models of response choice involve accumulation of evidence, in distributed brain regions, for each competing choice until decision thresholds are reached (Cisek, 2007; Glimcher, 2003; Rorie and Newsome, 2005; Smith and Ratcliff, 2004). Recent neurophysiological findings demonstrate that when there are two potential movement choices, initial activity within PPC neurons represents both potential targets, before one is suppressed and the other eventually dominates (Scherberger and Andersen, 2007), consistent with a mutual competition model of target selection (Cisek, 2006). From this perspective, competition between conflicting responses is a crucial process for action selection, analogous to models that propose competition to be a key part of selection for sensory attention (Desimone and Duncan, 1995; Duncan et al., 1997). Of course, the eventual response is likely to be based on the outcome of competition biased by many different aspects of an animal's state (e.g., previous experience, reward contingencies, and task-set) as well as changes in the environment (e.g., new information that alters the weight given to a particular stimulus).

Different brain regions might play distinctively different roles in situations of response conflict. While some may be the site of competition between responses activated by environmental stimuli, other brain regions might act selectively to enhance or reduce the impact of particular stimuli, for example, by applying “top-down” mechanisms (Corbetta and Shulman, 2002) to reduce the effect of information from flanker locations in the Eriksen task (Casey et al., 2000). The eventual RT would be the net result of influences on motor output from several brain regions involved in processing stimulus-response associations. We hypothesized that while the PPC might be involved in selection for action when stimulus-evoked responses conflict, frontal regions act to modulate the effect of irrelevant stimuli.

To investigate the possible role of the PPC in processing conflicting information for directional motor control, we tested four different groups of subjects with unilateral brain lesions on a modified Eriksen flanker task, with stimuli presented vertically in the midline to remove any confounding lateralized perceptual bias (Figure 1). We show that individuals with right PPC damage when cued to make rightward movements do not demonstrate the normal interference cost with incongruent (leftward) flankers. In fact, they show a highly paradoxical facilitation: they are actually faster to initiate rightward movements when there are leftward flankers (i.e., in the conflict situation) compared with when there are neutral flankers. In contrast, these individuals show clear RT costs for leftward movements in the presence of incongruent rightward flankers. Thus, their DH for leftward movements is most obvious in situations of response conflict, when rightward flankers interfere with movement preparation. These findings demonstrate that the PPC plays a key role during situations of response conflict. Moreover, its contribution to motor control may be most prominent when competition between alternative motor programs needs to be resolved. By contrast, data from individuals with more frontal damage show increased costs incurred in the presence of conflicting, flanker arrows, consistent with the proposal that these regions normally play a role in reducing the delays evoked by competing, irrelevant information in the stimulus environment.

In follow-up experiments using a free choice task (with no directional instructions) and a masked prime paradigm (where directional cues are not visible), we present further evidence supporting the proposal that the right PPC is a critical location for the automatic processing of leftward direction cues in situations of response conflict.

## Results

**Figure 2:**
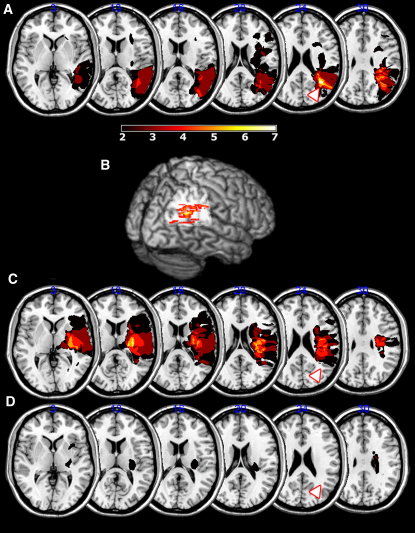
Comparison of Lesion Overlay for PPC/non-PPC Neglect Groups and Right Brain Damage Stroke Controls Area of maximum overlap in PPC neglect group in the angular gyrus shown both on axial slices (A) and 3D rendering (B). Non-PPC neglect patients had more anterior damage with a focus of overlap in the insular and inferior frontal white matter (C). Patients without neglect (non-neglect) had significantly smaller lesions, and they were more scattered throughout the right hemisphere (D). The white arrowhead with a red border points to the area of maximum overlap for the PPC neglect patients for comparison. Areas where two or more patients are affected are shown.

Using a central joystick, subjects made a speeded response leftward or rightward to a central target arrow flanked vertically by congruent arrows, incongruent arrows, or neutral shapes (Figure 1). The incongruent flanking arrows are normally considered to activate competing motor plans, thereby causing a delay in response initiation. In our task, the neutral cue consisted of a square symbol (made up of two of the arrows used as direction cues, but rotated such that they no longer carried any directional information). Seven patients with neglect, all with damage to the angular gyrus of the right PPC, were tested (Figure 2 and Table S1, available online), along with fourteen age-matched controls. All subjects were right-handed and used their right hands to perform the task.

### Facilitation for Rightward Movement in PPC Neglect Patients under Response Conflict

**Figure 3:**
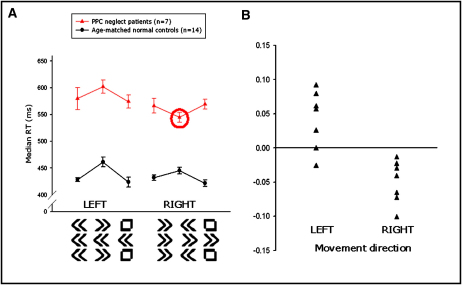
Reaction Times and Incongruence Costs in Patients with PPC Damage and Neglect (A) Median response times for PPC neglect patients and age-matched normal controls. Age-matched normal subjects show a reaction time cost in the incongruent (conflict) condition for both leftward and rightward movements. However, patients with PPC damage and neglect were all faster to move rightward in the incongruent condition than when the flankers were neutral or congruent. Error bars represent the standard error of the mean difference between incongruent and neutral RTs for incongruent and neutral RTs and the difference between congruent and neutral RTs for congruent RTs. (B) Corrected costs of incongruence (conflict) for all PPC neglect patients. Every patient with PPC damage and neglect was faster to move right when the flankers were incongruent than when they were neutral (or congruent; not shown here). All but one of the patients had a cost of incongruence for leftward movements. The one patient with facilitation for leftward movements had an unusual multifocal lesion due to carotid stenosis, but since it involved the right PPC and there was no evidence of any left hemisphere damage, he was included in the analysis.

In stark contrast to the performance of normal subjects, the neglect patients with PPC damage were actually faster in the incongruent (conflict) condition than the neutral or congruent conditions for rightward movements only (Figure 3A). Since there is variability in RT between the two groups, we calculated a corrected cost ([incongruent RT − neutral RT]/neutral RT) for each direction in each subject (Figure 3B). Repeated-measures ANOVA on the cost data for these two groups revealed a significant interaction between side and group [F(1,19) = 9.031, p < 0.01]. One-sample t test on the incongruence cost data for rightward movements of the “PPC neglect” group confirmed that there was significant facilitation (speeding) in the right incongruent condition (t = 3.226, p < 0.05). Thus, both groups—healthy controls and PPC neglect patients—had a cost for leftward movements on incongruent trials, but this cost in the conflict condition was lost in the PPC neglect patients for rightward movements. In fact, all patients with PPC damage were actually faster to move rightward when the flankers pointed leftward than when they were neutral; i.e., they had rightward incongruence facilitation rather than the normal cost in the conflict situation (Figure 3B). Importantly, response times to congruent and neutral flankers did not differ significantly in either direction for either group.

To our knowledge, no previous flanker study has shown speeding in the incongruent condition compared with a neutral one, in any subject group. We therefore sought an explanation within our data for this remarkable finding. First we asked if rightward facilitation could simply be the result of a generalized failure of patients with neglect and PPC damage to process leftward arrow stimuli. Paired-sample t test (uncorrected) on the median RT data showed no difference between leftward and rightward responses in the neutral or congruent conditions, making this an unlikely explanation (Figure 3A). We further considered the possibility that failure to decode leftward target-response associations might underlie the abnormality found in our PPC neglect patients (Bunge et al., 2002; Cavina-Pratesi et al., 2006), so we ran a free choice experiment on 24 stroke patients with neglect (Supplementary Material). In such experiments, when participants are free to choose their response, the two motor plans—leftward and rightward—are considered to be maximally in conflict because neither is favored by any external factors (Botvinick et al., 2001). This supplementary experiment revealed a rightward choice bias in patients with right PPC damage, even when there were no visual instruction signals to move left or right (Figures S4–S6, available online). Therefore, parietal neglect patients encounter difficulty preparing leftward movement plans when there is a competing rightward response, regardless of whether decoding a leftward visual target cue is required.

Attentional requirements and/or spatial selectivity (i.e., selection of responses directed by the central cue and not by the peripheral flankers) were also the same for both left and right incongruent conditions in our flanker task, so these factors cannot account for the directional facilitation we found either. However, attentional factors could explain the generalized slowing found in PPC neglect patients even though they were using their spared, ipsilesional arm (Figure 3). Such generalized slowing is a well-described finding, particularly in patients with right hemisphere damage, and may relate to failure of nonlateralized sustained attention (Howes and Boller, 1975; Husain and Rorden, 2003). Finally, note that participants made ballistic movements with the joystick without having to locate a spatially lateralized target. Therefore, pure attentional or visual localization accounts for directing movements to a visual target also cannot readily explain the directional difference found in our PPC neglect group.

Could a speed-accuracy tradeoff explain the rightward facilitation we observed in the response conflict or incongruent condition? If this were the case, one would expect that the error rate in the patients would be disproportionately raised in the right incongruent condition. However, this is not what we found. There were no significant differences in error rate between the flanker types in the PPC neglect group (Figure S1A). In contrast there were significant differences between conditions in the error rates in the normal control group (chi-square = 22.02, p < 0.001). Healthy subjects made significantly more errors in the incongruent condition, whereas, as a group, the PPC patients show only a nonsignificant trend toward this tendency, with some patients actually demonstrating the reverse effect (i.e., fewer errors in the incongruent condition). In addition, we investigated a speed-accuracy tradeoff by calculating an error cost of incongruence analogous to the RT cost described above (see Experimental Procedures). Again there was no suggestion of a raised error rate in the right incongruent condition (Z = −0.314, p = 0.753; Figure S1B). These error data rule out a speed-accuracy tradeoff as the cause for the speeded right movement in the incongruent condition. Rather, our data suggest a deficit at the level of motor preparation in PPC neglect patients that manifests when there is conflict between possible action plans.

Can rightward facilitation when there is such competition between action plans also account for any DH in PPC neglect patients? Note that in our group, there was no *directional* slowing in response initiation in either neutral or congruent conditions (Figure S2 and see above). However, these patients were significantly faster to move right than left only in the incongruent, conflict condition (t = 4.115, p < 0.01). These findings show that directional motor asymmetry occurs in patients with PPC damage, and neglect selectively, when there is competition between alternative responses. Importantly, this DH results *not* from leftward slowing but from rightward facilitation under situations of response conflict.

Next, we investigated whether rightward incongruence facilitation occurred only in neglect patients with right PPC damage. To do this, three further control groups were recruited. Right hemisphere stroke patients with neglect but without damage in the angular gyrus (“non-PPC neglect,” n = 7) were tested to establish whether rightward incongruent facilitation occurred in all patients with neglect or was lesion specific. We examined a second control group, this time “non-neglect” right hemisphere patients (n = 7), to see if deficits were neglect specific. Finally, left hemisphere patients (n = 7) with parietal damage were assessed to explore whether there were analogous abnormalities following left PPC damage.

### Neglect Patients without PPC Damage Have Increased Conflict Costs

**Figure 4:**
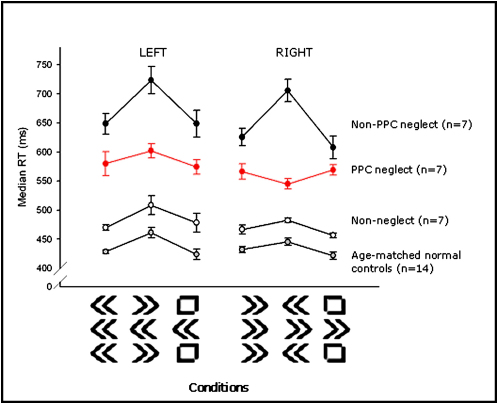
Reaction Times across Conditions All groups showed a reaction time cost in the incongruent (conflict) conditions for both leftward and rightward movements, except for the PPC neglect group, which demonstrated facilitation (faster RTs than neutral) in the conflict situation for rightward movements only (when the rightward cue was flanked by leftward arrows). Error bars represent the standard error of the mean difference between incongruent and neutral RTs for incongruent and neutral RTs and the difference between congruent and neutral RTs for congruent RTs.

Seven right hemisphere patients with neglect, but without any damage within the angular gyrus, were tested (Table S1). The area of maximum overlap for this patient group was within the white matter of the inferior frontal gyrus and insula (Figure 2C), distinctly different from the previous PPC neglect group. Apart from lesion location, non-PPC neglect patients were well-matched with the PPC neglect group in terms of age, lesion volume, and severity of neglect (independent samples t tests showed no significant difference between the two groups). Performance on the flanker task revealed that these non-PPC neglect patients did incur a cost of incongruence for rightward movements, unlike PPC neglect patients (Figure 4). Repeated-measures ANOVA on the cost data for the non-PPC neglect and the PPC neglect patients showed a significant interaction of group and side [F(1,12) = 16.223, p < 0.005]. The non-PPC neglect patients also differed significantly from normal controls in that they had a greater intrusion by incongruent flankers onto performance that was similar for both leftward and rightward movements [between subjects effect: F(1,19) = 5.891, p < 0.05; Figure 4].

Is there DH in this non-PPC neglect group? And is it affected by flanker type as in the PPC neglect group? We expected that neglect patients with more anterior damage might be more susceptible to visual distraction rather than a motoric initiation deficit as seen in the patients with PPC damage (Husain and Kennard, 1997; Mattingley et al., 1998). In our paradigm, the neutral flankers appear more visually arresting than the arrow flankers because they differ in form and are less frequent than arrows. Thus, any directional differences might be greatest in this neutral condition. (Evidence in support of neutral flankers having an arresting influence is provided in the Supplementary Material, where we report that normal subjects are significantly delayed [average 15 ms] when neutral square flankers accompany target arrows compared with when there are no flankers at all.) We found that non-PPC neglect patients were indeed significantly slower to move left than right only in the neutral condition (paired-sample t tests of leftward and rightward median RTs: t = 4.761, p < 0.005; Figure S2). Therefore, non-PPC neglect patients were particularly affected by the relatively unusual square flankers when planning a leftward movement. Taken together, the results suggest there might be two distinctly different forms of DH or motor initiation deficit: PPC neglect patients have relative facilitation of rightward movements during conflict, whereas non-PPC neglect patients with more frontal lesions demonstrate DH in the neutral, most visually arresting condition, with slowing of leftward movement initiation.

Given that in this non-PPC neglect group there were significant differences between left and right RTs in the neutral condition, a subsidiary analysis was performed to ensure that the bilateral difference in incongruence cost found between these PPC neglect patients and normal subjects was independent of directional difference in the neutral RTs. To do this we again calculated a cost of incongruence for each subject, but this time used the congruent RT as baseline ([incongruent-congruent RT]/congruent RT) because leftward and rightward congruent RTs did not significantly differ. Again there were significant between-group differences across both left and right movements [F(1,17) = 6.1, p < 0.05: average cost for normal subject = 5.2% (1.5%) versus non-PPC neglect = 14% (SE 2.8%)] and no significant interactions with direction of movement.

A further right hemisphere control group of patients without neglect was also tested and showed the normal flanker interference pattern (Figure 4 and Supplementary Material).

### Is There an Equivalent Effect of Lesions of the Left Hemisphere?

Seven patients with left PPC damage following stroke were also tested (Figure S3A) and used their left hands to perform the task (because of paresis of their right limbs). Their performance was compared with a different group of age-matched controls (n = 8) who also used their left hands. All seven of the left hemisphere group had lesions in the region of the angular gyrus of the PPC. Unlike the right PPC group, this left PPC group had a significant cost of incongruence for both leftward and rightward movements (Figure S3B). Repeated-measure ANOVA comparing the incongruence costs data from these left hemisphere patients with that of age-matched controls using their left hands showed no significant difference between the groups. Thus, patients with left PPC damage do not show the analogous deficit to those with right PPC damage when processing response conflict.

Four of these seven left PPC patients had apraxia when tested clinically, suggesting motor control deficits within the group (Table S1). Yet they displayed normal flanker interference patterns consistent with intact selection for action when two-directional responses compete. This implies different roles for left and right PPC in action selection, with left PPC patients manifesting difficulties in motor control when complex manipulations and functional object use are required (Buxbaum et al., 2006; Kawashima et al., 1993; Kimura and Archibald, 1974).

### Refining the Anatomical Locus Associated with Abnormal Response to Conflict

**Figure 5:**
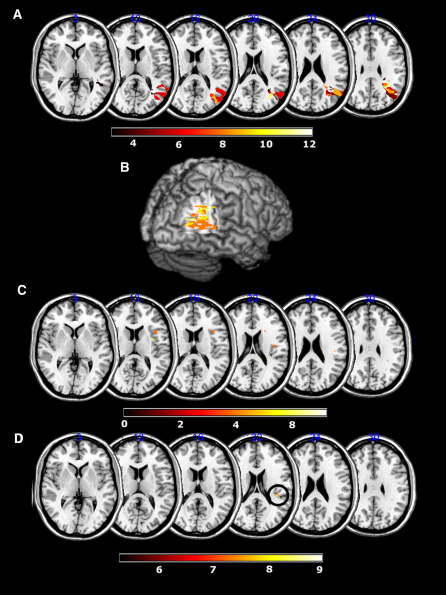
Lesion Loci Associated with Abnormal Performance (A) Damage to right angular gyrus was highly significantly associated with a reduced incongruence cost (facilitation). The highest Z score is 45 (Montreal Neurological Institute [MNI] coordinates of this area of damage: 38, −55, 24). Z scores over 4.62 are significant after Bonferroni correction at p < 0.05 level. (B) Three-dimensional rendering of the overlay showing the locations of areas significantly associated with a reduced or negative incongruence cost. (C) Right insula and inferior frontal gyrus damage significantly correlate with increased left incongruence cost. The highest Z score is 6.55 (MNI coordinates of this area of damage: 34, 14, 20). Z scores over 4.62 are significant after Bonferroni correction at p < 0.05 level. (D) Lesions of the right insula are significantly associated with leftward directional hypokinesia (black circle). The highest Z score is 9.05 (MNI coordinates of this area of damage: 34, −18, 20). Z scores over 4.62 are significant after Bonferroni correction at p < 0.05 level.

The data we have presented show that neglect patients with right PPC damage have a reduced incongruence cost for rightward movements, whereas patients without damage in the PPC tend to have increased RT slowing when movement cues conflict. Lesion overlap maps such as those in Figure 2 do not differentiate between loci of damage associated with abnormal behavioral performance and those areas most likely to be damaged by vascular insult in a particular territory. Therefore to investigate further the precise brain regions damaged in patients with a low rightward incongruence cost (or facilitation), we performed a permuted Brunner-Munzel rank order analysis on the continuous right incongruence, combining data from all 21 right hemisphere patients (PPC neglect, non-PPC neglect, and non-neglect; Figure 5A). The advantages of using the Brunner-Munzel rank order analysis are, first, that it is robust in the face of violations of normality and, second, the use of a continuous data set containing all three of our right hemisphere groups meant we were not required to divide the groups according to either lesion location or behavior prior to running the statistic (Rorden et al., 2007). Therefore this test provides a relatively assumption-free measure of whether or not damage at each voxel is associated with a reduced right incongruence cost (or facilitation by conflict).

Only voxels where three or more subjects had lesions were tested. Even after Bonferroni correction for multiple comparisons, right angular gyrus was the only area highly significantly associated with a low or negative rightward incongruence cost (Figures 5A and 5B). The most affected area lay within the cortex of the right angular gyrus, reaching a Z score of 45, with Z scores of >4.62 indicating a highly significant association with low rightward incongruence cost. This region of angular gyrus is just inferior to the intraparietal sulcus where neurons coding motor intention have been reported in monkeys (Andersen and Buneo, 2002; Stoet and Snyder, 2007). However, activations within the angular gyrus in humans during response conflict have been shown in fMRI studies (Botvinick et al., 1999; Liston et al., 2006). This discrepancy may reflect differences between human and monkey PPC. An alternative explanation is that the area identified in our analysis also includes white matter fibers, whose origins may include the intrapariatel sulcus.

Using the Brunner-Munzel rank order analysis, we next asked whether there was a brain region that, when damaged, rendered subjects more susceptible to irrelevant, competing stimulus-response activations (i.e., greater costs during conflict). We have already shown that a reduced right incongruence cost associates with PPC damage. Because all subject groups shared a positive conflict cost for leftward movements, we used the left incongruence data as a general measure of susceptibility to conflict. Again, all right hemisphere patients' scans were assessed (21 in total) and voxels affected in three or more individuals were probed to see if they were associated with a high leftward incongruence cost (Figure 5C). Brunner-Munzel rank order analysis revealed that anterior insula and inferior frontal white matter were both significantly more likely to be affected in those with an increased incongruence cost, again even after Bonferroni correction. The maximum Z score of 6.55 occurred in the inferior frontal gyrus (Z scores >4.62 being significant). Thus, damage to these frontal areas was associated with greater RT costs in situations of response conflict.

Lesion volume was also assessed as a possible predictor of patient performance. Lesions in the two neglect groups were larger than those in the non-neglect group (mean volumes: PPC neglect versus non-PPC neglect versus non-neglect = 9920 mm^3^ [SE 3515] versus 12024 [SE 2814] versus 2585 [SE 601], respectively). However, behavioral performance did not correlate with lesion volume either for the right incongruence cost (when all patients are tested together: Spearman's rho = 0.179, p = 0.439; or when just PPC neglect and non-neglect patients are compared: Spearman's rho = −0.446, p = 0.110) or for the left incongruence cost (all patients Spearman's rho = 0.123, p = 0.594).

### Which Brain Areas Are Associated with Leftward Slowing/DH?

Thus far the analyses have identified two types of DH: the first is relative DH due to rightward facilitation during conflict; the second DH, due to leftward slowing, occurs when flankers are neutral, suggesting that it is due to visual distraction during movement planning. Both conventional lesion overlap analysis (Figure 2A) and Brunner-Munzel rank order analysis (Figure 5A) show that the first type of DH is associated with right angular gyrus damage. Our final lesion analysis aimed to identify areas of the brain most likely to be damaged in any of the 21 right hemisphere patients who showed the second type of DH, reflected by a slower RT for leftward compared with rightward movements when flankers were *neutral*. Note that this is different from the analysis that examined brain areas associated with increased costs for leftward movements in the left incongruent condition (i.e., Figure 5C).

We ran a Brunner-Munzel rank order analysis, this time using the ([LEFT neutral RT] − [RIGHT neutral RT]) × (−1) as a measure of leftward slowing for each of the 21 subjects with right hemisphere damage. Even after Bonferroni correction, the area most associated with leftward directional slowing in the neutral condition was the right posterior insula (Figure 5D). The highest Z score was 9.05 (significance reflected by Z > 4.62). Therefore subjects with damage in the insula are significantly more susceptible to distraction from neutral flankers when planning leftward compared with rightward movements. Thus, both conventional behavior-lesion overlap correlation within each group and analyses performed on the combined data sets across all our right hemisphere patients provide a consistent pattern of results for this complex data set.

### Do Patients with Right PPC Damage Fail to Process Competing Leftward Motor Programs Activated by Invisible Primes?

Next we asked whether or not failure to process leftward action plans occurred even when conflicting information is not visually perceived. Masked primes activate response plans without the directional information reaching visual awareness (Eimer, 1999; Eimer and Schlaghecken, 1998). However, even though masked prime arrows are not visually perceived, they affect subsequent motor programming. The effect of prime arrows on a subsequent target is critically dependent on the interval between their presentation (stimulus onset asynchrony, or SOA) (Eimer, 1999; Eimer and Schlaghecken, 1998).

At short SOAs (<50 ms), congruent prime arrows (those pointing in the same direction as the target) speed response initiation to the target. However at SOAs of around 150–200 ms, there is paradoxical slowing of a target response when it is preceded by a congruent prime. This is the negative compatibility effect (Eimer, 1999; Eimer and Schlaghecken, 1998), which is the reverse of classical priming effects where congruent stimuli normally speed reactions. The negative compatibility effect is considered to result from automatic inhibitory mechanisms that prevent action on the basis of the irrelevant prime-induced response plans. The masked prime paradigm is considered to reflect competition between target directions because whenever one direction is inhibited, the other is facilitated, regardless of the SOA (Eimer, 1999; Sumner and Husain, 2007).

**Figure 6:**
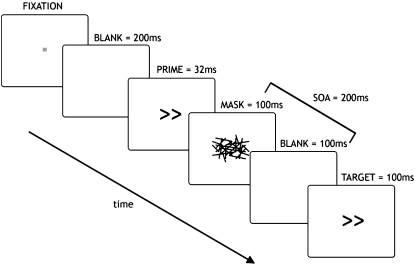
Masked Prime Paradigm Subjects respond to the target arrow using a centrally placed joystick. This arrow is preceded by a prime that is not visually perceived because it is masked. However, primes affect response times to targets. The stimulus onset asynchrony (SOA) used here is 200 ms (times between onset of mask and onset of target).

Here we tested 17 patients with right hemisphere stroke who responded leftward or rightward using a centrally placed joystick according to the direction of a target arrow presented 200 ms after a mask (Figure 6; Supplementary Material for patient details). The primes were neutral (squares), incongruent arrows, or congruent arrows.

**Figure 7:**
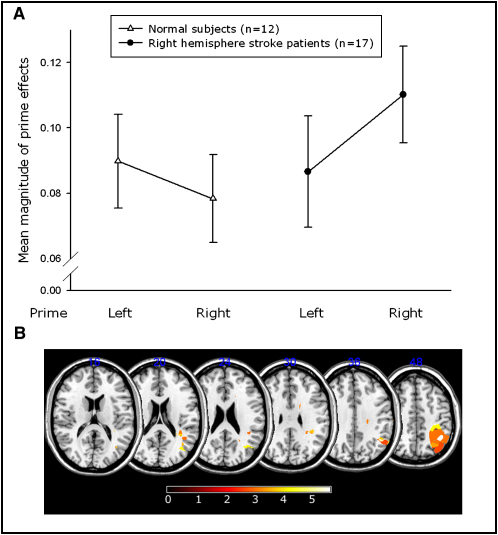
Effects of Left and Right Primes in Healthy Controls and Right Hemisphere Patients In contrast to normal subjects, the right hemisphere stroke group overall had a significantly lower magnitude effect of the left prime than the right prime (A). These data show the relative balance of the effects of directional primes. The magnitude effect for the left prime is calculated by adding together the magnitude of the prime effects for the conditions in which there was a left prime, i.e., left congruent condition ((|LC − LN|)/LN) plus right incongruent condition ((|RI − RN|)/RN). The calculation is similar for the right prime effects, but this time using analogous data from the condition in which the right prime was presented. Thus, the prime effects incorporate both leftward and rightward movements and control for possible directional effects in the patient group. Error bars represent the standard error of the group mean. Damage in the white matter underlying the angular gyrus and the supramarginal gyral white and gray matter is significantly associated with diminished processing of leftward relative to rightward primes (B). The coordinates for the most significantly affected area are x = 34, y = −52, z = 40 with Z scores of 5.77. Z scores above 4.72 are significant after Bonferroni correction (p < 0.05).

In contrast to normal subjects, the right hemisphere stroke group processed leftward primes significantly less effectively than rightward primes (Wilcoxon signed rank test, Z = 2.1, p < 0.05 in right hemisphere stroke patients compared with Z = 1.3, p = NS in normal subjects; Figure 7A). Note that in the masked prime task, inhibition of a prime, whether leftward or rightward, results in facilitation of the alternative movement direction. The prime effect measure we have used probes the relative balance between leftward and rightward motor programs. Hence it is the within-individual difference between left and right prime effects rather than the absolute magnitude of prime effects that is important. Thus, we make no claims about the difference between control and patient groups, but simply note that the balance between processing left and right primes in the patient group is biased against leftward direction cues.

Within the patient group, there was variability, with only some of the patients processing the leftward primes less effectively than rightward ones. Therefore, we asked if the difference between patients could be explained by lesion site. Specifically, on the basis of our previous experiments, we would expect that damage to the right PPC would be associated with a relatively reduced magnitude of left prime effects (Experimental Procedures). Using the Brunner-Munzel rank order analysis, we were able to confirm our hypothesis: damage only to voxels in the right PPC was significantly associated with a reduced left prime effect, as measured by the relative effects of left and right primes within subjects (Figure 7B; see also Supplementary Material). There was no significant clustering of voxels associated with reduced effects of right primes relative to left.

Taken together with the results of the Eriksen flanker task (where competing direction cues reach visual awareness) and our free choice paradigm (where there are no visual cues), this finding provides further independent evidence that competing leftward motor programs are processed selectively within right PPC, even when direction cues are not perceived but nevertheless compete automatically.

## Discussion

We used a modified Eriksen flanker task, with all stimuli presented in the vertical midline, to investigate how individuals with unilateral lesions process conflicting directional cues. Paradoxically, patients with neglect following right PPC damage were actually *faster* in the incongruent (conflict) condition than on neutral trials, but only for rightward movements. For leftward responses, they showed the normal pattern of RT costs in the presence of rightward flankers (Figure 3). To the best of our knowledge, focal lesion studies have not previously identified any brain region which, when lesioned, leads to direction-specific facilitation, as assessed by using the Eriksen protocol as a probe of response conflict (Ullsperger and von Cramon, 2006). Analogous effects were not observed following left PPC lesions. In contrast, neglect patients with right frontal damage were generally much slower and had a disproportionate increase in RT in the incongruent condition bilaterally. Thus, these individuals incur a significantly higher cost during response conflict, but this effect is not directionally specific.

We next examined response choices when participants were free to choose between moving left or right. Under such a condition, the two motor plans—leftward and rightward—are considered to be maximally in conflict because neither is favored by any external factors (Botvinick et al., 2001). We found that damage to the right PPC was significantly associated with rightward choices (Supplementary Material). Finally, we used a masked prime paradigm, which measures the effects of invisible direction cues (primes) on subsequent responses. This paradigm probes competition between target directions, even when such competition is evoked subliminally (Sumner and Husain, 2007). On this independent measure, we found that leftward directional primes have reduced effects, relative to rightward primes, specifically in patients with right PPC damage (Figure 7).

How can we account for such a reversal of the RT cost (facilitation) from incongruent flankers in our PPC neglect group? Careful analysis of our data excluded several possible explanations. First, we asked if the neglect patients with right PPC damage simply did not process the leftward arrow stimulus normally, i.e., if they experienced difficulty recognizing leftward arrows as a signal to move left and therefore took longer to respond. This is made highly unlikely by the finding that when a leftward target arrow was presented centrally with neutral or congruent flankers, these individuals were able to initiate leftward responses with similar latencies to rightward movements. In addition, patients with right PPC damage displayed a rightward choice bias even in the absence of visual cues requiring decoding but when leftward and rightward plans are considered to be maximally in conflict (Supplementary Material).

Next we considered an explanation based on visual attention, i.e., that perhaps PPC neglect patients had an exaggerated, narrow “spotlight” of attention on the central target and thus automatically filtered the flanker information more than normal individuals (Casey et al., 2000). However, the attentional requirements were identical for both leftward and rightward movements, and it is evident that a cost was incurred in the leftward incongruent condition, but not the right. Thus, the abnormal finding was unidirectional, precluding an explanation based solely on attention. Could rightward conflict facilitation in the PPC neglect group reflect a spatial deficit for left-sided stimuli? We specifically designed our flanker task to exclude the possible confound of spatial bias: all stimuli were presented in the vertical midline and subjects responded by using a central joystick to make ballistic movements without needing to identify or localize a lateralized visual target. In our view, therefore, none of these accounts—perceptual, attentional, or spatial—explain our findings, although it is important to stress that this does not mean that abnormalities in each of these domains do not occur in neglect patients.

We propose that direction-specific facilitation in the incongruent condition in PPC neglect patients is best explained by a motoric deficit that occurs selectively when response plans compete. Specifically, leftward flanker-induced response plans do not appear to be activated commensurately in neglect patients with right PPC damage when they are in competition with a target-induced rightward response plan. Such limited inhibition of rightward movements may effectively lead to over “impulsivity” toward the right in situations of response conflict in these individuals.

### Independent Parietal and Frontal Responses to Conflict

**Figure 8:**
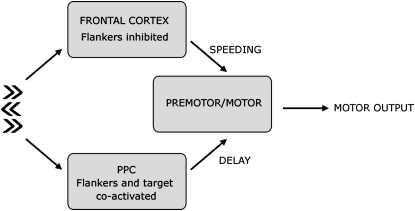
Schematic of Interaction between Parietal, Prefrontal, and Premotor Regions in Response Selection When two responses conflict, such as in the incongruent condition of the Eriksen flanker task, both possible responses (evoked by target cue and flankers) are activated within the parietal lobe. These responses mutually inhibit one another, causing response delay. In contrast, the frontal cortex enhances the target and/or inhibits the flankers selectively to speed response initiation. Each of these areas influences the decision threshold reached in the premotor cortex. Damage to the PPC reduces the response delay, but the intact prefrontal cortex still boosts the target response, thus producing the facilitation observed in our PPC neglect patients.

But while failure to represent response alternatives in PPC neglect patients might explain loss of the incongruent RT cost, remarkably, our patients with parietal damage actually showed significant facilitation for rightward movements in the presence of leftward flankers, i.e., faster responses than in the neutral condition. To account for the paradoxical facilitation observed in this group, we invoke the action of a second system, this time in the frontal lobe, which we suggest acts simultaneously with the PPC (Botvinick et al., 1999, 2001, 2004; Carter et al., 1999; MacDonald et al., 2000). In our schema, PPC and prefrontal regions interact with premotor or motor regions to influence response choice and movement initiation when conflicting responses compete for selection (Figure 8).

According to our proposal, part of the response delay in situations of response conflict such as the flanker task is attributable to competition between partially activated stimulus-evoked response associations (for target and flankers) within the PPC. In contrast to the parietal role, prefrontal cortex may selectively enhance target information, inhibit flankers, or both. Indeed, previous imaging and behavioral work suggests that prefrontal cortex may selectively suppress flanker-induced (or irrelevant prime-induced) activity and enhance target processing purely on the basis of the rule that information at flanker locations is irrelevant to the task goal (Casey et al., 2000; Egner and Hirsch, 2005; Sumner et al., 2007). Thus, these more anterior regions potentiate the target response and hasten the initiation of movement in the direction of the dominant motor plan (target arrow in the flanker task).

Critical to our proposal is the idea that PPC and prefrontal responses are activated independently by the stimulus conflict in the environment (target versus flankers in our experimental situation). In patients with damage to the PPC, the incongruence delay is lost, or at least greatly reduced, because competition between partially activated stimulus-evoked response associations is reduced. But the key point is that prefrontal regions remain intact in these individuals. They therefore continue to enhance the response to the target and/or inhibit the response to the flankers, regardless of whether the PPC is “off-line.” Without any competition in the (lesioned) PPC, which normally contributes to the RT delay, the net result of such prefrontal activity would be relative facilitation, i.e., response times that are faster than neutral, as we observed in our PPC neglect patients. Conversely, when prefrontal regions are damaged, the delay due to competition within the PPC still occurs, but now without the potentiation (speeding) of target responses from more anterior areas. Thus, incongruent responses are disproportionately slow as in our neglect patients with anterior white matter and insula damage (see Supplementary Material and Figure S8 for further details of how such facilitation might emerge with independent parietal and prefrontal systems).

It is evident, however, that the facilitation we observed in our right PPC neglect group was only for rightward movements (with leftward flankers). Is there a system in the left parietal lobe mirroring processes occurring in the right PPC? Functional imaging studies have demonstrated bilateral parietal activation during response conflict tasks (Bunge et al., 2002; Cavina-Pratesi et al., 2006; Liston et al., 2006). However, behavioral data from our left hemisphere patients did not reveal any directional-specific facilitation. One possible explanation for this is that the right PPC has a bilateral function in resolving motor competition, whereas the left hemisphere fulfils a unidirectional role promoting competing rightward movements only. This would be analogous to the bilateral allocation of spatial attention within the right inferior parietal lobe, thought to explain the higher incidence of unilateral neglect following right hemisphere strokes compared with left hemisphere strokes (Heilman and Vandenabell, 1980; Mesulam, 1981). Therefore, the right PPC compensates to some degree for the loss of the left PPC in our left hemisphere patients, but the reciprocal compensation after right PPC damage cannot occur.

It is important to note that patients with right parietal and frontal lesions are still able to make leftward and rightward movements with the right hand, but that there are distinctly different abnormalities of motor programming in these two patient groups. The different findings in these groups suggest that, normally, parallel processing streams must be activated and be capable, to some extent, of independent activity. Recent evidence suggests these processing streams may influence motor output via a final common pathway involving premotor cortex (Figure 8), where information on action choices accumulates until a decision threshold is reached (Cisek and Kalaska, 2002, 2005; Glimcher, 2003; Passingham, 1993). Note that although we consider parietal and frontal systems to be activated independently by response conflict, we would not suggest that interactions between these systems do not normally occur; clearly, there are massive parieto-frontal connections that mediate such traffic. Our scheme simply proposes that regions within the PPC and frontal lobe may be activated in parallel by response conflict, and that damage to one system does not preclude activation in the other.

### Directional Motor Deficits in Parietal and Frontal Neglect

Finally we consider the directional motor deficits found in neglect patients and their anatomical localization, a topic that has been highly controversial (Bartolomeo et al., 1998, 2001; Coulthard et al., 2006; Harvey, 2004; Heilman et al., 1985; Mattingley et al., 1998; Mesulam, 1999; Sapir et al., 2007). Slowing of leftward movement initiation, or DH, has been reported following both parietal and frontal lesions (Mattingley et al., 1992, 1998). In our study, we have shown two clear-cut patterns of DH, both of which could exacerbate the neglect syndrome. Neglect patients with PPC damage had directional imbalance due to rightward facilitation only in the incongruent condition when competing response plans are activated. Thus, the directional deficit in PPC neglect was due to faster rightward responses (i.e., relative leftward slowing) when there was conflict or competition between motor plans, analogous to models that propose competition to be a key part of selection for sensory attention (Desimone and Duncan, 1995; Duncan et al., 1997).

In contrast, neglect patients with damage in the right posterior insular demonstrated DH only in the neutral condition, which is most visually arresting, suggesting that these individuals struggle to filter the intrusive effects of visual distraction when planning a leftward compared with a rightward movement. Moreover, the DH in these cases was due to the slowing of leftward responses, not the speeding of rightward ones as in the PPC patients. Thus, the experiments reported here have demonstrated a dichotomy between parietal and more anterior neglect patients in their directional response speeds depending on the context of the instructed movements. This dissociation may explain the heterogeneity in previous studies, which have reported patients grouped according to their clinical syndrome without necessarily distinguishing between cases according to their lesion anatomy.

#### Conclusion

Previous studies of conflict-related brain activity have largely focused on the way the brain acts to minimize intrusion from (unwanted) conflicting information. However, in our view, for optimal control over behavior it is critical for conflicting information (cueing alternative movement plans) to be processed and evaluated before an action choice is made. Here we have identified a system involving the PPC that activates competing motor plans in response to conflict and may underlie the response delay observed when we respond to incongruent information. Recent findings from recordings in monkey PPC also demonstrate that when there are two potential movement choices, initial activity within these neurons represents both potential targets (Scherberger and Andersen, 2007), consistent with a mutual competition model of target selection (Cisek, 2006). The interaction between this parietal system, which we consider to play a key role in competition for action selection, and the prefrontal system, which may limit interference on performance, may be essential for flexible control of behavior in an environment that presents rapidly changing situations of response conflict.

## Experimental Procedures

### Participants

Patients were recruited from stroke and neurological units with local ethics committee approval. Initially we tested seven stroke patients with neglect and damage in the angular gyrus of the PPC (mean age: 64; range: 41–78). This is the PPC neglect group. Then we recruited three control groups: seven neglect stroke patients without damage in the angular gyrus of the PPC (mean age: 66; range: 36–67), which we refer to as the non-PPC neglect group; seven non-neglect right brain damage controls (mean age: 62; range 31–80; two tumor excisions; five stroke); and seven stroke patients with left hemisphere damage (mean age: 59; range: 44–68); see Table S1 for patient demographics. All subjects with right hemisphere stroke used their right hands to perform the task and those with left hemisphere stroke used their left hands, because some patients had contralesional hemiparesis.

Fourteen age-matched normal control subjects completed the conflict task using their right hands (mean age: 57.8; range: 23–76). Subsequently, eight further normal controls performed the task using their left hands to act as controls for the left hemisphere patients, who also used their left hands due to the high incidence of right hemiparesis. All subjects tested were right-handed and gave written consent according to the Declaration of Helsinki.

### Behavioral Assessments

Visual neglect was assessed using a battery of tests (Parton et al., 2004). All patients diagnosed with neglect showed behavioral neglect in everyday activities and also showed neglect on the Bells cancellation task and/or line bisection on 17 cm lines. Neglect was identified by rightward asymmetry of three or more targets found on the Bells cancellation task or a rightward line bisection deviation of 5 mm or more.

### Apparatus and Stimuli

Subjects moved a custom-built Traxsys (Ringwood, UK) desktop joystick with a 6 cm pole. The position of the joystick was polled every 10 ms. The joystick was fitted with a spring that automatically centered the pole. Deadspace was 1% of total movement (0.45°). Stimuli were presented in the vertical midline using Presentation (Albany, USA) software on a Sony Vaio laptop (PCG-5A1M) for 200 ms (Figure 1). Interstimulus interval was 2 s after initiation of the response. Arrow stimuli were designed so that the directional information would be available to patients even if they had a hemianiopia or object-based neglect; each arrow comprised two chevrons pointing in the same direction and subtended approximately 3 × 2° visual angle. Neutral cues comprised the arrows rearranged so that they carried no directional information; they formed a square (Figure 1).

### Conflict Task

Subjects sat approximately 100 cm away from the 15” laptop display and were required to move the joystick as fast as possible leftward or rightward in response to centrally placed arrows. Above and below the target arrow were flankers (separated from target arrow by approximately 3°) that were congruent, incongruent, or neutral (Figure 1). The stimuli were randomly presented with the constraint that each condition appeared the same number of times per block. There were eight blocks, each containing 24 trials, giving a total of 32 trials per condition (six conditions). A short practice session (<2 min) took place before the start of the first block. Subjects were instructed to keep their gaze on the laptop display and eye position was monitored by the experimenter. It was explained that there were no visual targets for them to aim for and that they should move the joystick as quickly as possible to its end-stop (25 mm lateral movement).

#### Data Analysis

Initially we compared six conditions (incongruent, congruent, and neutral flankers for rightward and leftward movements) in neglect PPC patients and normal controls. Median RTs were used because RT data tended to be positively skewed, particularly in the patient groups. This limited the amount of data trimming to only response times less than 200 ms (anticipations) and greater than 1500 ms; responses with these RTs were excluded from any part of the analysis. The higher boundary was >3 standard deviations away from any individual's average RT and reflected trials where the subjects had failed to respond without instruction. The program only moved into the next trial after a response from the subject to ensure that the patients were alert throughout.

Congruent and incongruent RTs were compared with neutral RTs and expressed as a cost or benefit, which was then converted to proportion of the neutral RT to account for differences in response time between the groups (i.e., [median incongruent RT − median neutral RT]/median neutral RT). Repeated-measures ANOVAs were performed separately on the cost and benefit data, which fulfilled criteria for parametric statistics (response direction as a within-subject factor, and subject group as a between-subject factor). In order to investigate the nature of DH, paired-sample t tests comparing leftward and rightward median RTs for each condition in each group were carried out. Since all PPC neglect patients appeared to perform faster in the right incongruent condition, a single-sample t test was performed on the incongruence cost data with zero as the reference sample to see if there was significant facilitation within this group.

Error data was not normally distributed, and therefore nonparametric statistics were used for the analysis. Friedman test was applied to the proportion of errors for leftward and rightward movements in congruent, incongruent, and neutral conditions. In addition we calculated an error cost of incongruence analogous to the RT cost of incongruence described above for leftward and rightward movements ([error_incon_ − error_neu_]/error_neu_; see Figure S1B). Since several subjects made no errors, in order to carry out this calculation, we transformed the data by adding a single extra trial considered to be half error and half correct (Snodgrass and Corwin, 1988); the total number of trials in each condition was increased by 1 (approximately 3%) and the total number of errors was increased by 0.5 (approximately 1.5%).

Data were nonparametrically distributed (Shapiro-Wilk test, p < 0.05). Since we were concerned with ruling out the possibility of significantly increased error rate selectively for the right incongruent condition in patients with PPC neglect, we performed an uncorrected Wilcoxon signed rank test on the error data despite the risks of obtaining a false positive result. This showed no suggestion of a directional difference, and even when incongruent minus congruent errors are considered ([error_incon_ − error_con_]/error_con_), there is no difference between left and right error rates within the PPC neglect groups (Wilcoxon signed rank score = 0, p = 1).

Similar analyses were carried out for each control group. Repeated-measures ANOVAs were used to compare each control group with the PPC neglect group, and a subsidiary analysis compared the controls groups with the age-matched control group. Further t tests were used to investigate specific hypotheses regarding directional speed differences.

### Lesion Plotting

Lesions were plotted from routine clinical CT or MR scans (9 MR, 7 CT) onto a standard CH2 template using MRICro software available at www.mricro.com. Overlays and 3D renderings were carried out in MRICron (www.sph.sc.edu/comd/rorden/mricron/) after conversion of regions of interest (ROIs) to voxels of interest (VOIs). Permuted Brunner-Munzel rank order analysis was performed on the right incongruent cost and lesion data for the whole stroke group using MRICron software and nonparametric mapping (NPM for windows also available from www.mricro.com). Only areas affected in at least three individuals were included in the analysis. Bonferroni corrections were performed automatically using the MRICron NPM software. Further lesion analyses again used the Brunner-Munzel rank order analysis and all 21 stroke patients, but different behavioral data; left incongruence data ([left incongruence cost]^∗^[−1]) was used as a measure of general susceptibility to incongruence and (left − right median RT)^∗^(−1) for each individual was the index used for investigation of leftward movement slowing.

Lesion volume was estimated using MIPAV software (Centre for Information Technology, Bethesda, MD). Lesion volumes were then compared between groups using independent samples t tests and correlated with behavioral performance where appropriate.

### Masked Prime Task

#### Participants

Seventeen patients with right hemisphere stroke (twelve neglect; Table S3) and twelve age-matched healthy controls (seven female, average age 63.5 years) were recruited. All subjects were right-handed and used their right hands to perform the task.

#### Apparatus and Experimental Paradigm

Patients were positioned approximately 100 cm from a 15” Sony Vaio (PCG-5A1M) laptop screen where stimuli were presented centrally using Presentation (Albany, USA) software (Figure 1).

Initially subjects fixated a central box that disappeared 200 ms prior to prime onset (Figure 6). The prime was presented for 32 ms (two screen refreshes) and was followed immediately by a mask consisting of 30 randomly oriented lines that was presented for 100 ms. Then a blank screen was shown for 100 ms before a target arrow was presented for another 100 ms (i.e., SOA = 200 ms). Subjects were required to respond as fast as possible to the target arrow using a centrally placed joystick and were instructed to keep their gaze on the laptop display. With an SOA of 200 ms, many previous experiments have shown that there is an RT cost, or negative compatibility effect, when the prime and target point in the same direction compared with when they point in opposite directions or when the prime is neutral (no directional association) (Sumner, 2007).

Masking rendered the prime imperceptible. To ensure the prime had been successfully masked, all subjects were asked to describe what they saw after the first block and, at the end of the experiment, subjects were asked if they saw any arrows other than the ones following the hashed lines, and none did. Intertrial interval was 2 s after initiation of the response. Eye position was monitored by the experimenter. A short practice session (<2 min) took place before the start of the first block.

Each arrow comprised two chevrons pointing in the same direction and subtended approximately 1.5 × 1° visual angle. Neutral primes comprised the arrows rearranged so that they carried no directional information; they formed a square (not shown) and covered the same area as the arrow stimuli.

There were 12 blocks of 24 stimuli and stimulus presentation was randomized with the constraint that each condition occurred the same number of times per block. There were six different trial types.

#### Data Analysis

Repeated-measures ANOVA was performed on raw median RT data for each subject group separately and also jointly with subject group as a between-subjects factor and direction and prime type as within-subjects factors. Post hoc pairwise comparisons were performed with Bonferroni correction where appropriate (see results in the Supplementary Material).

Previous work has suggested that elderly people who respond slowly have a reduced negative compatibility effect (Schlaghecken and Maylor, 2005). Therefore, to control for differences in RT and possible generalized failure to process masked primes, two transformations are made to data for subsequent analysis. First, reaction time differences are expressed as proportions of the neutral RT for each individual, and second, only lateralized differences (in prime direction) are considered.

The hypothesis was that patients with PPC damage would propagate leftward directional programs less well than rightward ones. Therefore the magnitude of the effect of the left prime compared with the right prime was the subject of the next investigation. The magnitude effect of the left prime was calculated as:

((|LC−LN|)/LN)+((|RN−RI|)/RN)

and the right prime was calculated as:

((|RC−RN|)/RN)+((|LN−LI|)/LN)

where RC = median right congruent RT, RI = median right incongruent reaction time, RN = median right neutral reaction time, LC = median left congruent RT, LI = median left incongruent RT, and RN = median left neutral RT.

Thus, the effect of left primes is calculated from all conditions in which a left prime is presented, and vice versa for right primes.

The magnitude of the prime effects was calculated for each stroke patient and normal subject. The data were nonparametrically distributed, and therefore Wilcoxon signed rank test was performed for both groups comparing right with left prime effects. The difference between the left and right prime effects for each individual (L − R) was used to identify patients with relatively reduced processing of the left prime. Spearman's nonparametric correlation was performed to investigate any relationship between reduced leftward prime effects and neglect severity using the Bells cancellation score (R − L cancellations; for results, see Supplementary Material).

#### Lesion Mapping

All patients' lesions were plotted using MRICro software (available at www.mricro.com) with routine clinical imaging, either CT or MR, on the CH2 template to create an ROI on the axial images at Z coordinates 56, 61, 66, 69, 75, 85, 88, 92, 96, 102, 108, and 120. Brunner-Munzel rank order analysis (NPM for windows, www.sph.sc.edu/comd/rorden/mricron/) was used to establish which areas were associated with relatively reduced magnitude of left prime effects. The statistic was calculated only at regions where three or more subjects were affected, and Bonferroni correction was applied (postcorrection significance level of p < 0.05). Lesion volume was calculated for each patient using MIPAV software (version 4.0.1, NIH, Bethesda, MD) and correlation was sought between lesion volume and relative impairment of left prime processing using Spearman's rho because lesion volume data were not normally distributed (for results, see Supplementary Material).
