# Supplementary material for: Control over Conflict during Movement Preparation: Role of Posterior Parietal Cortex
Source: Neuron. 2008 Apr 10;58(1):144–57. doi: 10.1016/j.neuron.2008.02.009 (PMC2292819; doi:10.1016/j.neuron.2008.02.009)
Supplement: Document S1. Supplemental Material [file mmc1.pdf]

Neuron, volume 58

Supplemental Data

Control over Conflict during Movement Preparation: Role of Posterior Parietal Cortex

Elizabeth J. Coulthard, Parashkev Nachev, and Masud Husain

Table S1 Clinical information for patients performing modified Eriksen flanker task

	Age (years)	Time since stroke (months)	Bells R-L cancellation score	Line bisection (mm to the right)
PPC Neglect right hemisphere				
	73	94	4	24
	78	1	14	20
	41	0.1	3	5
	66	0.1	1	6
	41	0.3	13	0
	42	0.2	4	9
	64	0.3	5	8
Medians:	**64**	**0.3**	**4**	**8**
Neglect non-PPC right hemisphere				
	36	0.2	0	7
	67	1.8	4	22
	66	0.1	10	7
	64	0.2	7	7
	66	0.3	3	4
	66	0.7	4	10
	64	0.8	14	4
Medians:	**66**	**0.3**	**4**	**7**
Non-neglect right hemisphere				
	74	0.7	-1	-4
	31	0.1	0	0
	62	1	-1	-1
	70	0.2	0	0
	53	0.3	1	-2
	80	0.6	1	0
	57	60	0	2
Medians:	**62**	**0.3**	**0**	**0**
Left hemisphere				
	68*	26	-2	1
	58*	1	0	9
	69*	24	-2	-2
	34	28	1	-4
	68*	90	-1	-2
	64	0.3	0	0
	62	25	-2	-2
Medians:				

* = evidence of significant apraxia on copying gestures or miming actions

Patients without neglect have normal response costs in the flanker task

Next we asked if right hemisphere patients without neglect had either reduced or increased costs of incongruence. We tested 7 further right hemisphere patients, now with no evidence of neglect either in terms of symptoms or performance on standard clinical tests **(Table S1 and Fig 2d)**. As with all other control groups, **non-neglect** patients did not show facilitation by conflict for rightward movements (repeated measures ANOVA on the cost data for PPC and non-neglect patients revealed a significant interaction of group x side ($F(1,12)=4.895$, $p<0.05$) revealing again that PPC neglect patients are distinctly different). The non-neglect patients did not significantly differ from normal controls in their rightward or leftward incongruence costs **(Fig 5)**, and neither did they show any significant directional difference in RT or DH **(Fig 6)**.

Free choice experiment

Introduction

In this additional experiment we examined whether the failure to represent competing leftward motor plans observed in right-hemisphere neglect patients was truly due to a motor deficit, or whether these individuals have problems decoding visual stimuli with a leftward response association. We devised a choice task, in which on half the trials subjects were asked to select between making leftward or rightward movements as quickly as possible; whereas on the other 50% of trials they were instructed which way to move **(Fig. S1)**.

The stimulus to freely choose left or right comprised two squares which carried no directional information. Therefore, there are neither rightward nor leftward *visual* cues in this condition. Nevertheless, in this ‘underdetermined’ response situation, the two motor plans **-** leftward and rightward **-** are considered to be maximally in conflict because there are only two alternatives and neither is favored by any external factors (Botvinick et al 2001).We hypothesized that patients with right parietal damage would have a bias toward making rightward movements under such free choice conditions because leftward motor plans would not compete equally with rightward ones.

Methods

Twenty-four patients with right hemisphere stroke (**Table S2 -** not all the same as in the conflict task: 16 neglect, 4 recovered neglect and 4 never neglect) and 14 normal controls performed the task. Importantly, this group of patients was not divided according to lesion location or the presence of neglect in advance. Rather, using specific predictions from the

conflict task above, we hypothesized that a rightward bias (tendency to move right more often than left) would be associated with right PPC damage when continuous data from all patients were considered. A subsidiary analysis is also presented where patients are divided a priori according to whether or not they have PPC damage, regardless of whether or not they have neglect **(Fig S3a).** Three of the four recovered neglect patients had damage within the PPC.

Again, subjects moved a centrally placed joystick left or right as quickly as possible. Stimuli similar to those used in the conflict task, presented in the vertical midline, were either congruent arrows all pointing in the same direction (*instructed* condition; 50% of trials) or neutral flankers with no central target (*choice* condition; remaining 50% trials); see **Fig. S1**. On choice trials, subjects were instructed to move as fast as possible either left or right. They were explicitly told to move with their first instinct and not to make a plan in advance about their direction of movement. Instructed cues were included to make it harder for subjects to form a plan in advance about which way to go. Stimuli were presented pseudo-randomly with the two constraints that each condition appeared the same number of times per block and that the same stimulus was presented no more than twice in a row. Four blocks of 48 trials were performed giving a total of 196 trials. A short practice session (< 2mins) took place before the start of the first block.

Numbers of responses leftward and rightward (degree of lateralized bias) and median RTs left and right are reported in the free choice and instructed conditions. Again we performed the Brunner-Munzel rank order statistic on the continuous behavioral (((number R **-** number L)/ (number R + number L)) x 100 x **-**1) and lesion data to show areas selectively affected in patients with a rightward bias. For comparison we also compared the performance of patients who had damage within the PPC compared with those who did not have significant damage within this region.

Results

Overall, stroke patients moved rightward 51% of the time (SE 2.8%) and normal subjects moved rightward 49% of the time (SE 2.1%), but there was considerable variation between individuals, particularly those with right hemisphere stroke, with some showing a bias to make rightward movements and others selecting leftward movements more often (**Fig S2**). In the subsidiary analysis, there was a significant difference in percentage of rightward responses chosen between those patients with PPC damage and those without ($t = 2.3$, $p<0.05$: PPC patients went right 57 % (SE 2.8) of the time on average compared to 46% (SE 4.0) in the patients without PPC damage).

Parietal damage was significantly associated with a rightward bias

Brunner-Munzel rank order statistic revealed that relative rightward bias in the neglect patients was significantly associated with damage to the cortex and white matter of the angular gyrus of the inferior parietal lobe ($p<0.05$ after Bonferroni correction **-** red areas in **Fig S3a**).

Reaction time analysis

Neither the stroke patients nor the normal controls showed any directional speed differences in the instructed condition **(Fig S4)**. This is important because one could argue that despite the central position of the stimuli and the joystick, rightward response bias in patients with right hemisphere stroke was related to a covert attentional bias toward the right. However, if this were true, one might expect speeding towards the right (or slowing towards the left) in the instructed condition. This is entirely in keeping with our hypothesis from the conflict experiment. Since the congruent flankers used in the instructed condition were neither visually distracting nor did they activate competing response plans, directional slowing we would be expected in either parietal or frontal patients. Analysis of response speed in the choice condition would be confounded by frequency of response choice and was not performed. Interestingly all groups showed a non**-**lateralized increase in reaction times when responses were chosen rather than being instructed ($F(1,36)=69.7$, $P<0.001$) and this is cost of choice is significantly larger in patients with stroke (instruction/choice X subject group $F(1,36)=17.1, p<0.001$).

Discussion

The proximity of the parietal zone associated with rightward bias to that found in the previous experiment supports the proposal that individuals with right PPC damage favor rightward over leftward motor plans when the two are in competition. Since this directional bias occurs in the absence of any visual leftward signal, it reflects impairment of a pure directional motor process which normally occurs within the PPC.

The performance of patients without neglect overlapped with those who manifest neglect on standard clinical tasks. However, there was a significant association of *lesion location* with relative rightward bias. This suggests that such a rightward motor response bias is directly related to lesion location rather than the presence or absence of neglect. Although it is quite possible that right motor choice bias exacerbates neglect symptoms.

Not all patients with PPC damage chose to go right more often than left. For example, of the 6 PPC neglect patients who showed facilitation in the conflict task, only 4 went right more

often than left. According to our framework we would expect a relative rightward bias more in those patients with parietal damage compared with other groups. However, this prediction is tempered by the knowledge that multiple factors may combine to influence response choice including perhaps an individual's decision to try to move left and right an equal number of times. Although we encouraged subjects to move as fast as possible and not to make a plan, it is not possible to rule out some other sort of decision processes influencing their movement choices. Therefore we expected, and found, overlap between PPC, non-PPC and normal controls groups in the proportion of rightward movements chosen. Since we included a large number of patients in our lesions analysis, we were still able to find significant association of PPC damage with rightward response bias.

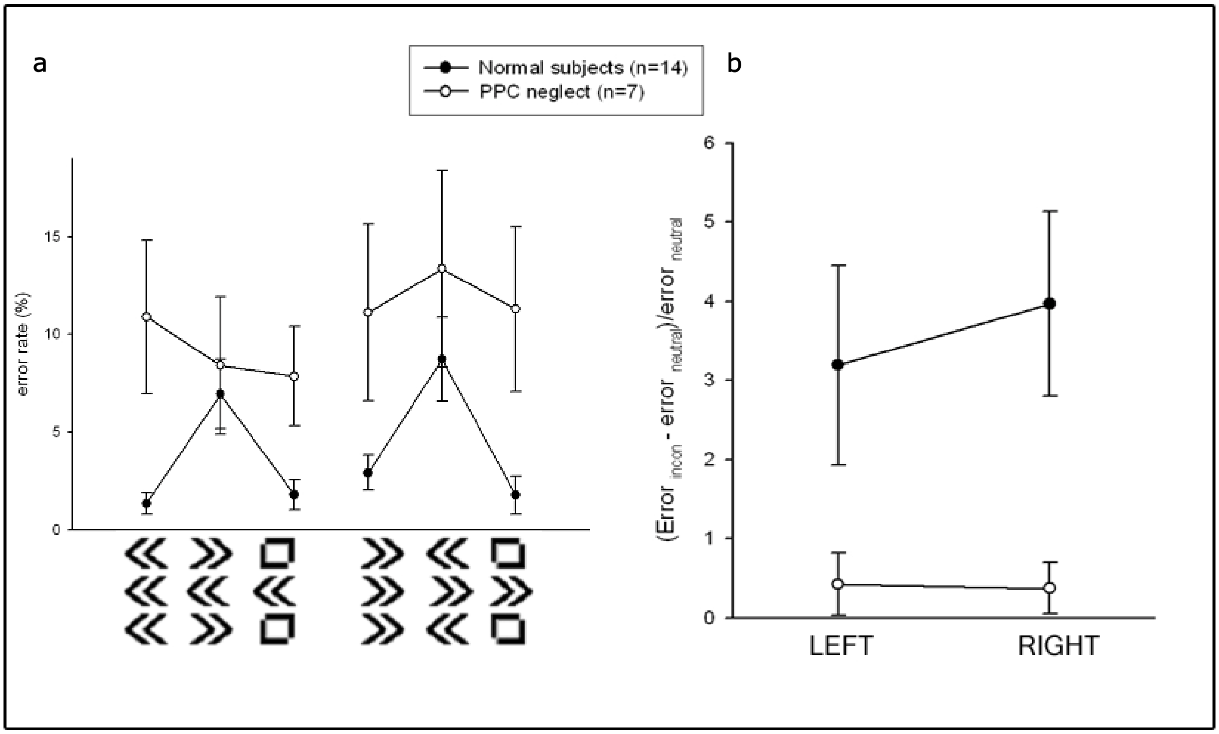

Figure S1 Error rates in normal control subjects and PPC neglect patients

a) Normal subjects have increased errors in the incongruent condition. Even when the error cost of incongruence is calculated (b), there is no trend for right PPC neglect patients to show an increased error rate for rightward incongruent compared with leftward incongruent movements. Note that although error rates appear higher in the right than left incongruent condition, this effect is not consistent, with 3 of 7 PPC neglect subjects showing the reverse pattern.

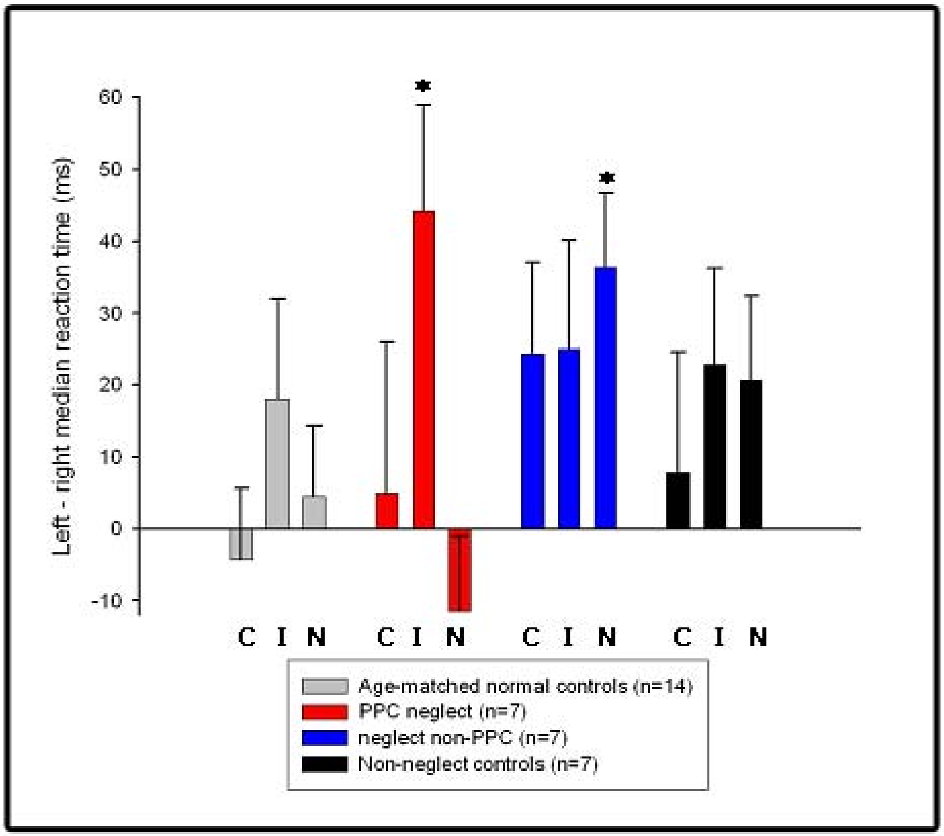

Figure S2 Directional hypokinesia for each flanker condition
PPC neglect patients were significantly slower to move left than right, but only in the incongruent condition when there are competing motor plans. Non-PPC neglect patients were significantly slower to move left than right, but only in the neutral condition which was visually most distracting. C: congruent; I: incongruent; N: neutral.

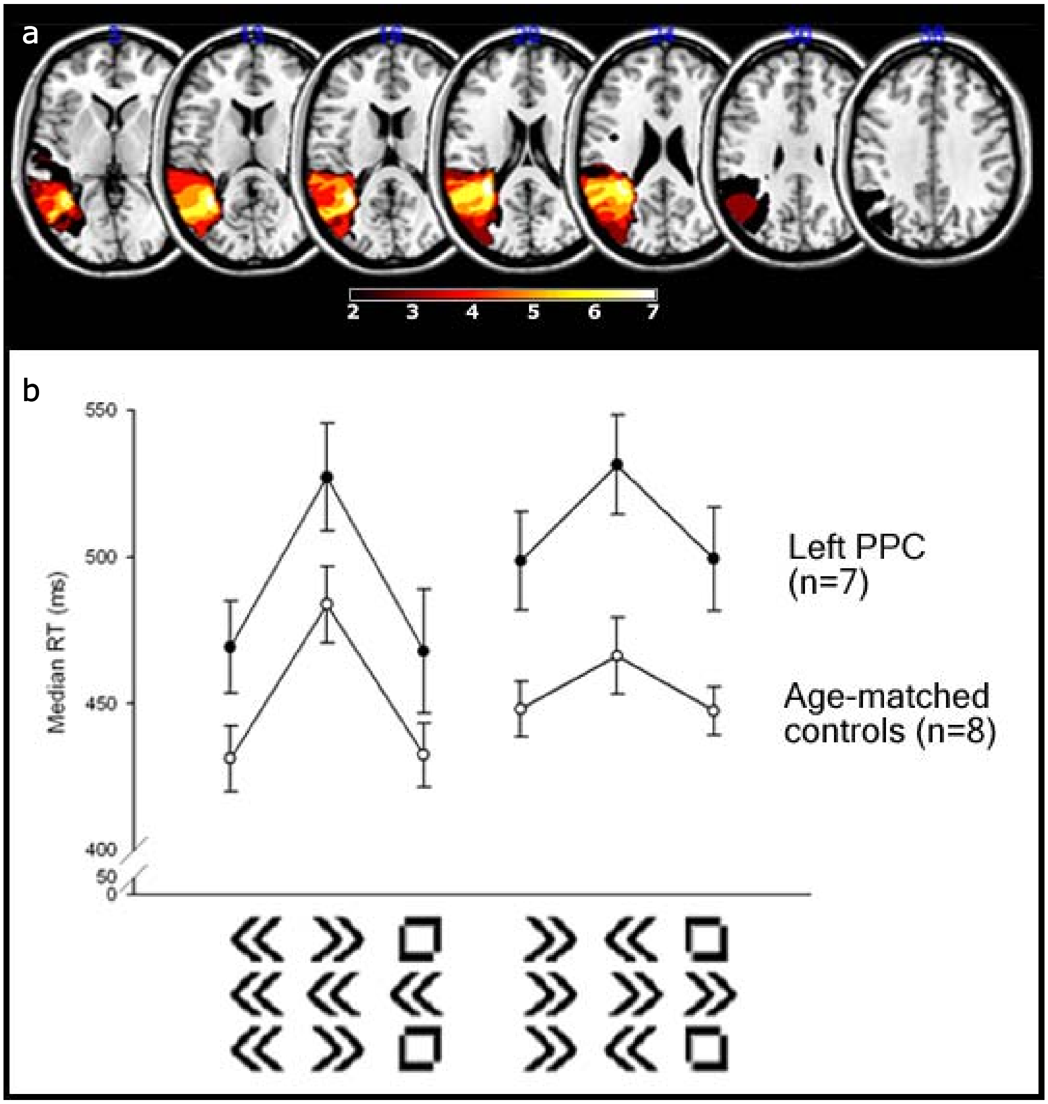

Figure S3 Lesion overlay and behavioural performance for left hemisphere patients.
Three of the subjects had damage in the angular gyrus of the left PPC (a). Left hemisphere patients were slower in the incongruent condition for both leftward and rightward movements (b).

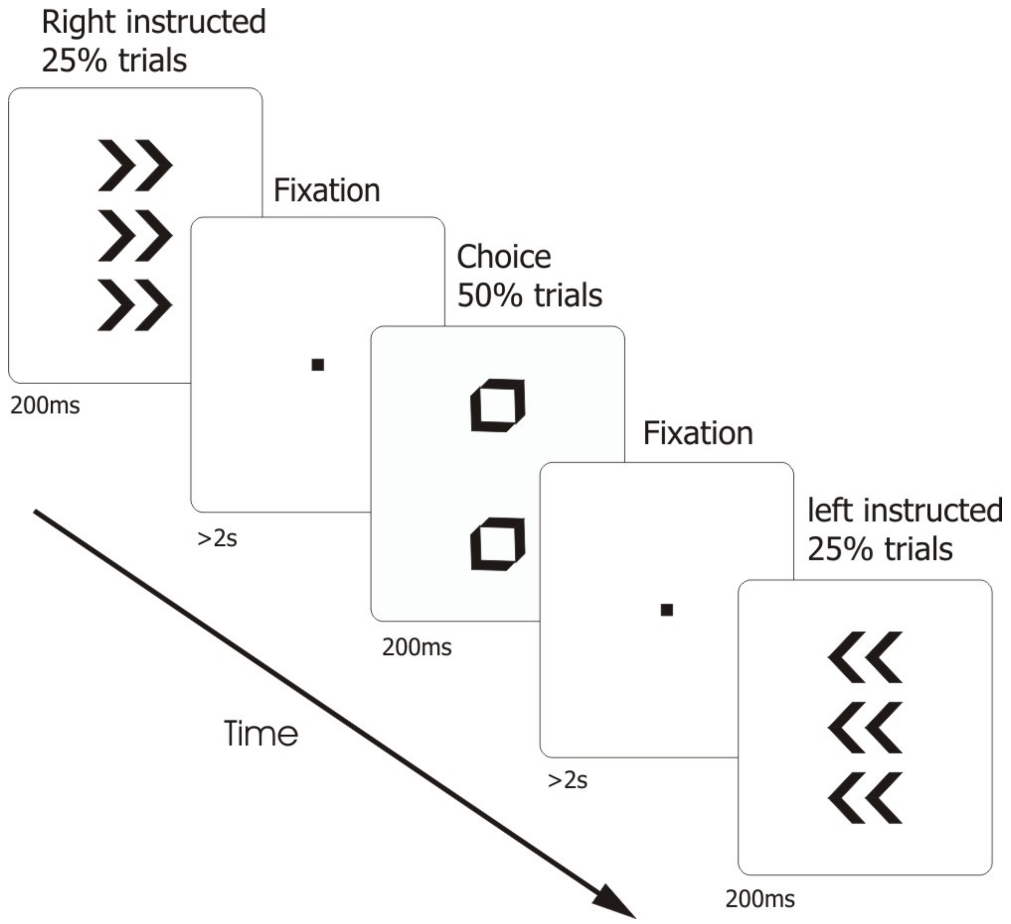

Figure S4 Free choice task
Participants had to choose to move either left or right as fast as possible on choice trials (50%). In the other 50% of trials, subjects were instructed to move either left or right by arrow cues. Trials were pseudo-randomised with the constraint that the same number of each trial type occurred in each block and that no more than 2 free choice trials in a row were presented.

	Age (years)	Time since stroke (months)	Bells R-L	Line bisection (mm to the right)	Current (neg), recovered or never neglect
Neglect					
	78	1	14	20	neg
	58	35	3	-2	neg
	41	0.06	3	5	neg
	66	0.09	1	6	neg
	41	0.3	13	0	neg
	42	0.2	4	9	neg
	64	0.3	5	8	neg
	36	0.2	0	7	neg
	47	1.5	1	20	neg
	67	1.8	4	22	neg
	66	0.09	10	7	neg
	66	0.25	3	4	neg
	66	0.68	4	10	neg
	46	1.5	14	28	neg
	69	1.13	3	8	neg
	46	0.68	8	18	neg
Median	61	0.5	4	8	
Non-neglect					
	74	0.68	-1	-4	never neg
	31	0.09	0	0	never neg
	24	2.58	0	0	recovered
	63	58.87	0	4	recovered
	70	0.16	0	0	never neg
	53	0.19	1	-2	never neg
	67	0.58	1	3	recovered
	71	2.32	0	4	recovered
Median	65	0.6	0	0	

Table S2 Patients for Free choice task

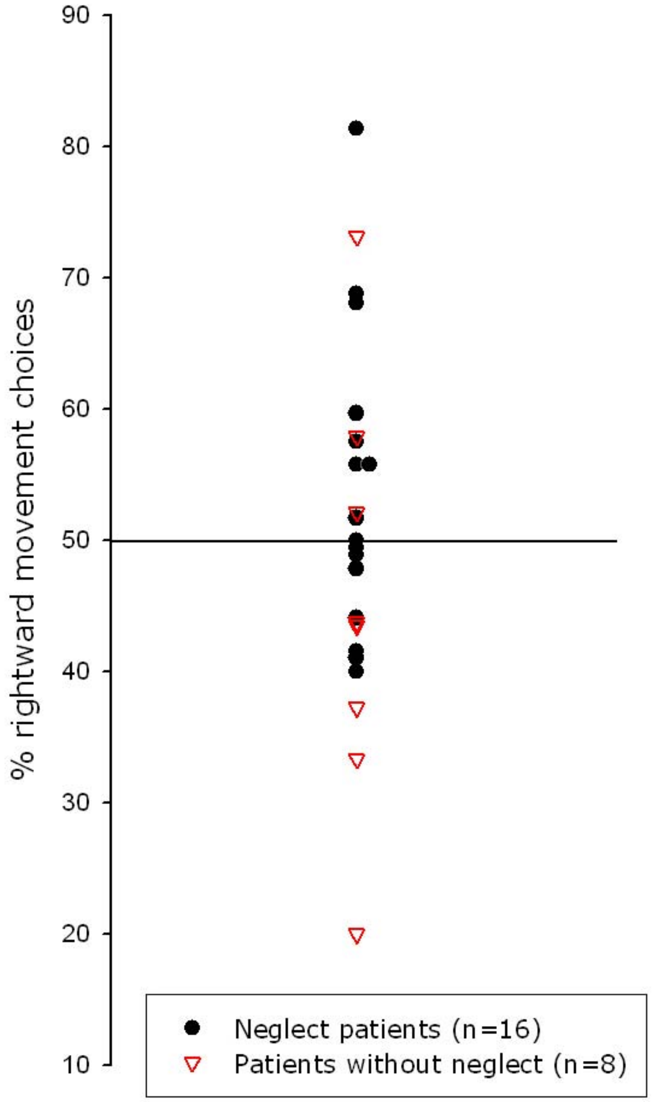

Figure S5 Proportion of rightward responses across groups
In the choice task, subjects chose to respond left or right on 50% of trials. Positive values indicate a rightward bias and negative values suggest a leftward bias. There was great variability in performance with this right hemisphere stroke group, but note that performance for neglect and non-neglect patients overlapped.

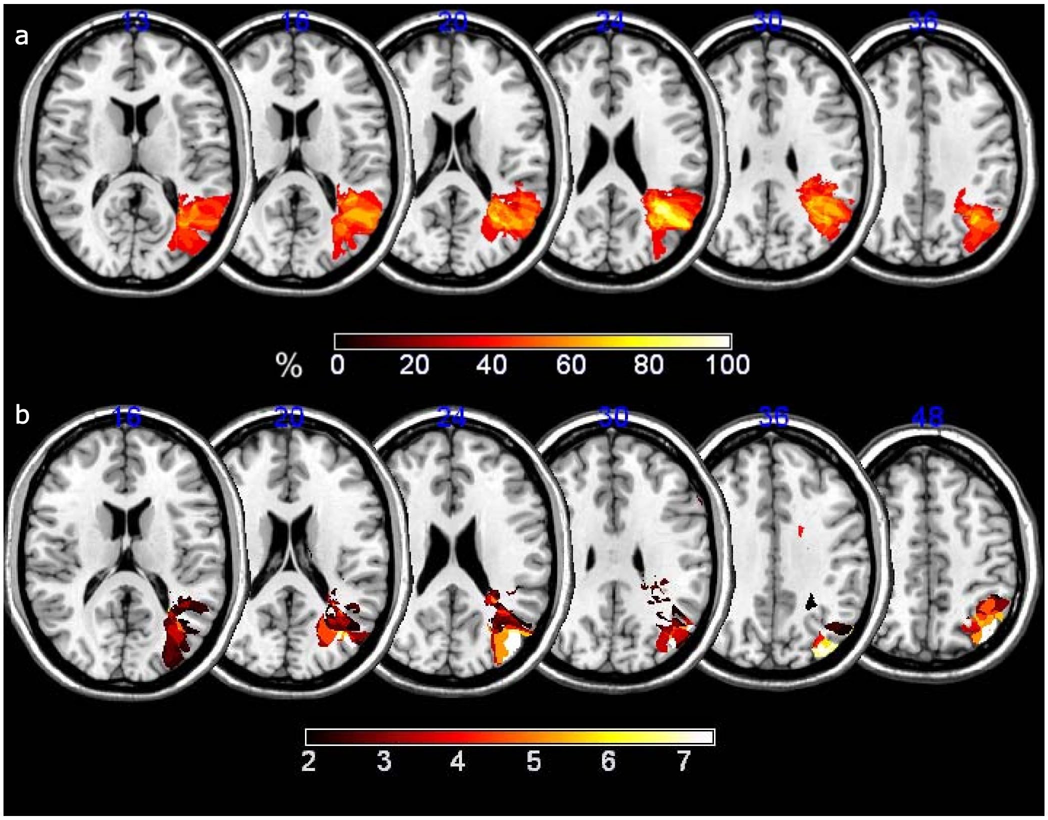

Figure S6 Lesion analysis for neglect patients in free choice task
a) Subtraction showing areas damaged in patients with lesion of the PPC (patients with PPc damage minus those without). Colour bar shows the percentage damaged in PPC group and not in non-PPC group e.g white colour shows that 100% of PPC patients had damage in this area and 0% of non-PPC patients.

b)Using the Brunner Munzel rank order statistic, cortex of the angular gyrus and surrounding white matter are shown to be significantly associated with a rightward bias, even after Bonferroni correction for multiple comparisons ($p<0.05$). The coordinates of the centre of the significant area on this slice are x=50, y=-64, z=24. A Z score of greater than 4.745 suggests significance after Bonferroni correction.

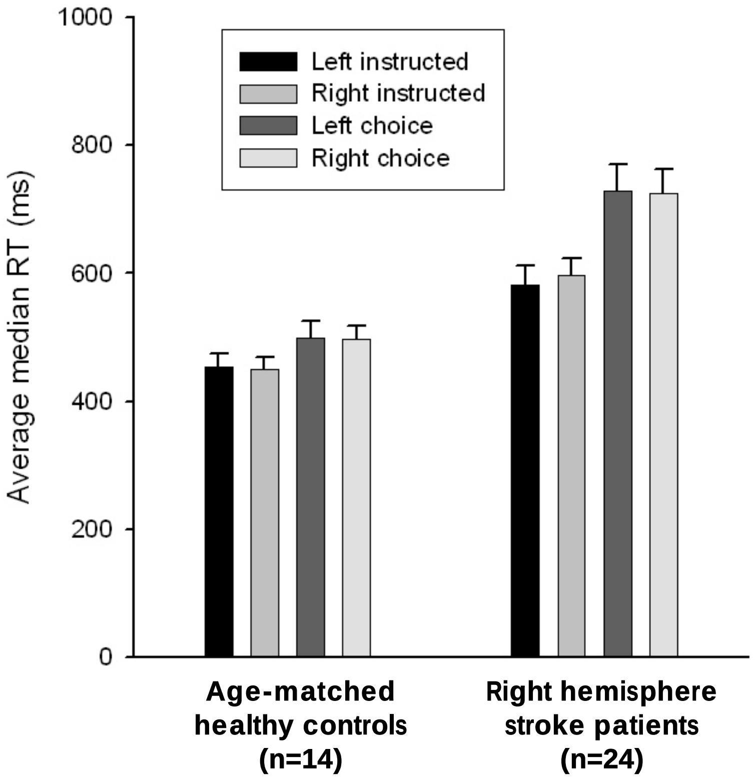

Fig S7 Average median RTs for normal controls and right hemisphere stroke groups
All subjects were slower when they had to choose to move (either right or left) rather than being instructed. Note that no group showed any directional speed differences. Even a subgroup analysis of those right hemisphere stroke patients who had neglect on the day of testing did not suggest that there was any leftward slowing – choice condition RTs: R= 773ms (SE = 50); L = 774ms (SE = 50ms).

Visual distraction by neutral flankers

In this experiment, we investigated the visual distraction by neutral flankers in six normal subjects. The setup was exactly as for the conflict paradigm except that either left or right target arrows were presented alone (2/3 of trials) or in association with neutral, square flankers (1/3 of trials). The proportions were chosen to mimic exactly the proportion of neutral trials in the conflict experiment. 300 trials in total were performed (50% right. 50% left).

Median RTs were calculated for each individual in the no flanker and the neutral flanker conditions. Paired sample t-test on this data revealed a significant delay in the neutral compared with the no flanker condition ($t=4.7$, $p<0.005$). The average delay was 15ms with 4 of the 6 subjects having a within-subject significant delay in the neutral flanker condition.

This finding provides evidence that when neutral flankers are presented as in the conflict paradigm, they cause delay despite not carrying any conflicting response association. Therefore, the delay must be due to sensory factors such as visual distraction. The result is in keeping with left directional slowing in the neutral condition only in patients with anterior lesions being due to visual distraction particularly when leftward movements are planned.

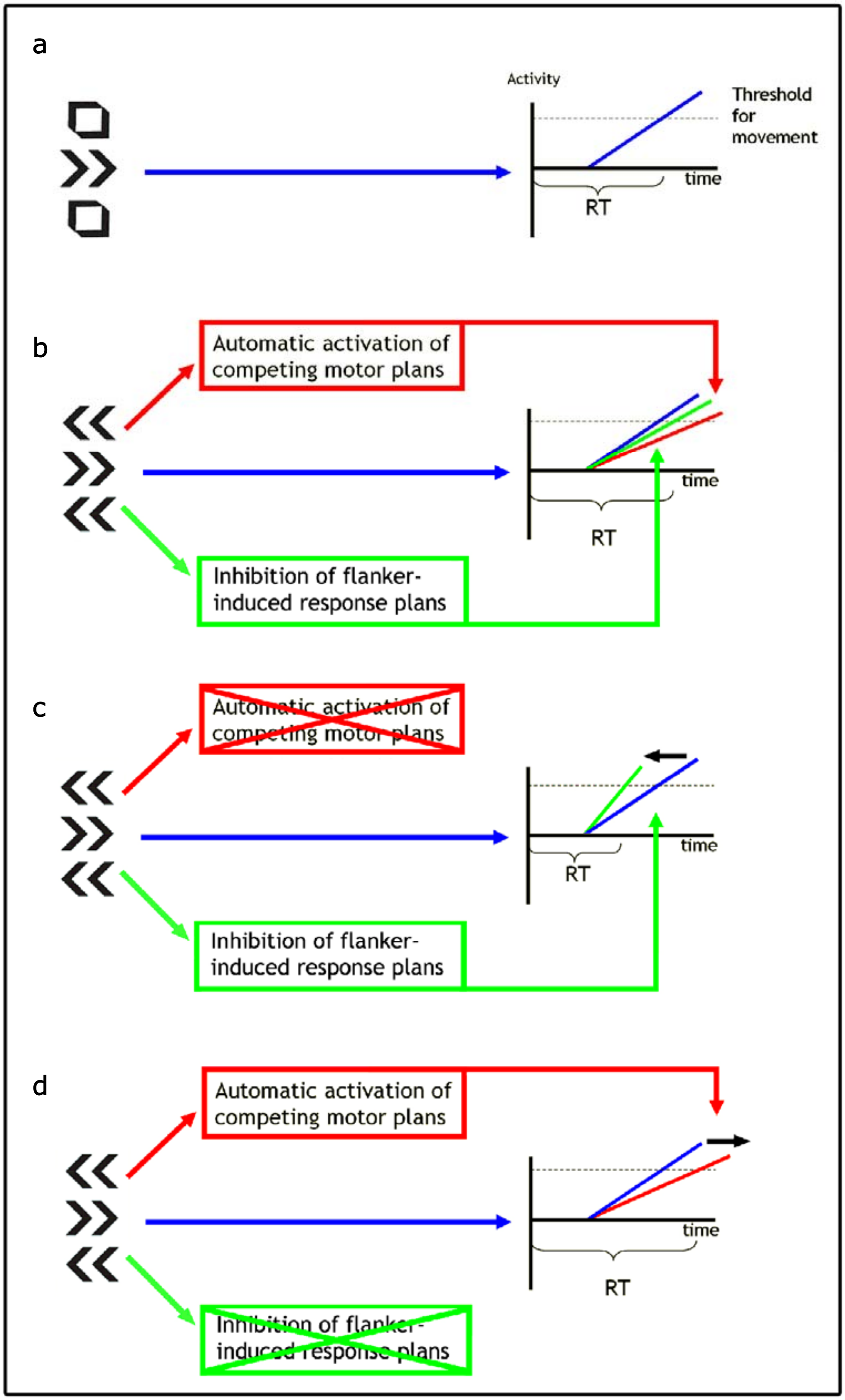
a
Activity
Threshold for movement
time
RT
b
Automatic activation of competing motor plans
time
RT
Inhibition of flanker-induced response plans
c
Automatic activation of competing motor plans
time
RT
Inhibition of flanker-induced response plans
d
Automatic activation of competing motor plans
time
RT
Inhibition of flanker-induced response plans

Figure S8 Schematic of parietal and frontal systems activated by conflict

a) Response when there is no conflict. Blue line shows hypothetical rise in neural activity to a threshold level for motor initiation (dashed horizontal line) b) When the flankers are incongruent, the activation of competing motor plans in the parietal lobe leads to a delay in response (red line). This delay is minimised by prefrontal regions which actively inhibit unwanted flanker-induced response plans. The reciprocal effect of this flanker inhibition is enhancement of the target response. The net result of these two systems, parietal and frontal, is a small delay compared to the neutral condition (green line). c) When there is parietal damage, automatic activation of motor plans does not occur and there is no delay. However, the intact prefrontal system still enhances the target response and there is facilitation of response compared with the neutral condition – the pattern observed in our patients with parietal damage. d) In the case of damage to prefrontal areas or their connections, there is automatic activation of alternative plans in the intact parietal lobe and consequent delay. But in this case, the systems which normally increase inhibit flanker-induced responses are damaged. So, one would expect a longer delay in the incongruent condition such, as observed in our patients with frontal lesions. It is important to note that we have represented frontal and parietal alterations in neuronal activity as changes in the ramp up to movement threshold (slope of the line). However, it is equally possible that the threshold for movement is changed by these areas. Our data do not distinguish these two possibilities.

Table S3 Clinical information for patients in masked prime task

Age (years)	Time since stroke (months)	Bells R-L cancellation score	Line bisection (mm to the right)
Neglect			
77	6	3	-2
46	2	3	6
76	99	4	8
67	4	8	12
66	0.4	1	5
61	1	8	9
41	2	4	8
68	2	12	10
45	1	7	10
39	2	10	18
63	1	3	1
Median 62	2	4	8
Non neglect			
68	7	0	-2
66	1	0	1
42	0.5	-2	0
57	2	2	4
67	3	2	4
56	48	2	4
Median 61.5	2.5	1	2.5

Supplementary results for masked prime paradigm

Normal subjects did have the negative compatibility expected at an SOA of 200ms (main effect of prime $F(2,22) = 14.9$, $p < 0.005$, **Fig S6).** However, there was no significant effect of the prime in the right hemisphere stroke group, as expected given that we were expecting lesion-specific effects described in the main text.

Reduced magnitude of the left prime effect was shown to be associated with PPC damage (see main text). We also asked if reduction in the left prime magnitude could be associated with the presence of neglect. Spearman non-parametric correlation did not suggest a correlation between neglect severity and reduced processing of the left prime (Spearmans rho = 0.261, NS). Thus the effect is lesion-specific rather than neglect-specific.

We also considered whether lesion volume could contribute to the prime effects. However, there was no suggestion of correlation between the size of the lesion and the relative diminution of left prime effects (Spearmans rho = -0.047, NS).

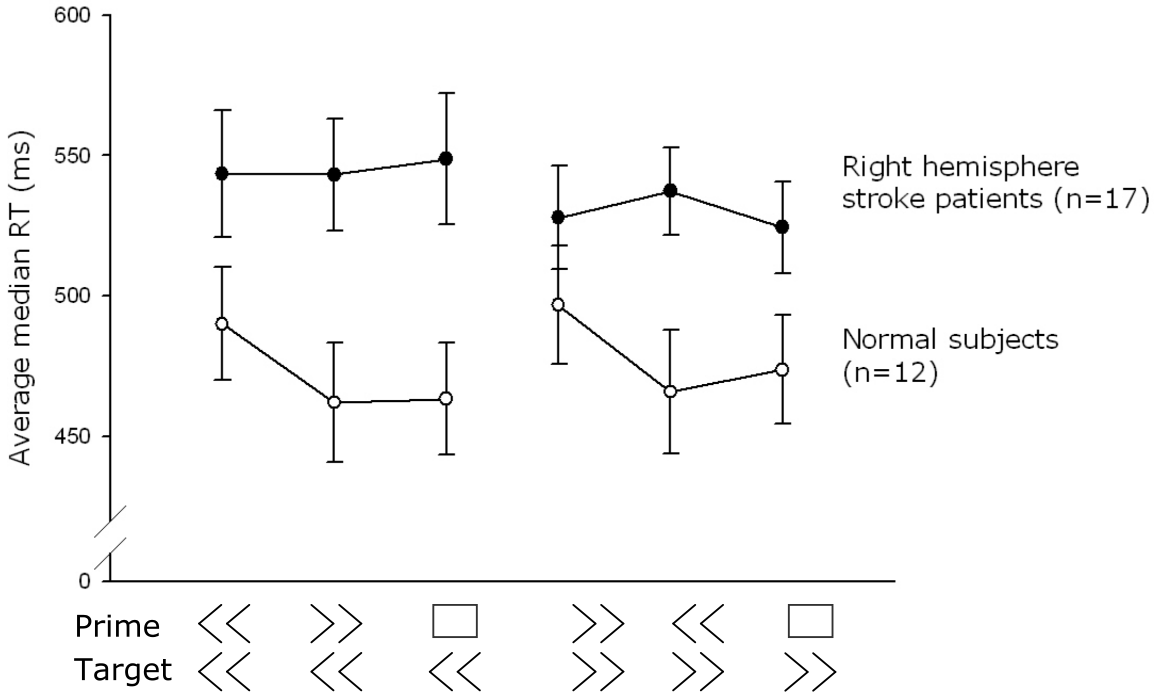

Figure S9 Median RTs for normal controls and right hemisphere stroke subjects
Normal subjects are significantly slower to move either leftward or rightward when a congruent prime precedes the target, i.e. they manifest the negative compatibility effect at this SOA of 200ms. There was variability within the patient group with no significant prime effects when the group was considered as a whole.
